# Turn up the healthy eating and activity time (HEAT): Physical activity outcomes from a 4-year non-randomized controlled trial in summer day camps

**DOI:** 10.1016/j.pmedr.2020.101053

**Published:** 2020-01-14

**Authors:** Keith Brazendale, Michael W. Beets, R. Glenn Weaver, Gabrielle M. Turner-McGrievy, Justin B. Moore, Jennifer L. Huberty, Dianne S. Ward

**Affiliations:** a University of Central Florida, Department of Health Sciences, Orlando, FL, USA; b University of South Carolina, Department of Exercise Science, Columbia, SC, USA; c University of South Carolina, Department of Health Promotion, Education and Behavior, Columbia, SC, USA; d Wake Forest School of Medicine, Department of Family and Community Medicine, Winston-Salem, NC, USA; e Arizona State University, School of Nutrition and Health Promotion, Phoenix, AZ, USA; f University of North Carolina at Chapel Hill, Department of Nutrition, Chapel Hill, NC, USA

**Keywords:** Physical activity, Intervention, Children, Summer camp

## Abstract

Summer day camps (SDCs) serve over 14 million children in the U.S. and are well-positioned to help children accumulate the guideline of 60 min per day (60 min/d) of moderate-to-vigorous physical activity (MVPA). The purpose of this study was to evaluate a multi-component intervention to increase the percentage of children meeting 60 min/d of MVPA. Twenty SDCs serving 3524 children (7.9 yrs., 46.2% girls, 66.1% non-Hispanic Black) participated in a 4-summer non-randomized two-group intervention. Children’s accelerometer-derived MVPA was collected using accelerometers worn on the non-dominant wrist. SDCs were assigned to either 2 summers of intervention (n = 10, no intervention summer 2015, intervention summer 2016 and 2017) or 1 summer of intervention (n = 10, no intervention summer 2015 and 2016, intervention summer 2017). The final summer (July 2018) was a no intervention follow-up. Multilevel mixed effects regression models estimated changes in percent of children meeting 60 min/d of MVPA. Across all summers and SDCs, children accumulated an average of 89.2 min/day (±22.5) of MVPA. The likelihood of meeting the 60 min/d MVPA guideline was not different during intervention versus baseline summers for boys or girls (p > 0.05). Girls and boys were 3.5 (95CI = 1.5, 8.1) and 3.7 (95CI = 1.6, 8.4) times more likely to meet the 60 min/d guideline during intervention summers versus follow-up, respectively. The intervention was not successful at increasing the percentage of children meeting the 60 min/d MVPA guideline. However, children attending SDCs accumulated substantial amounts of MVPA thus efforts should focus on making SDCs an accessible setting for all children.

## Highlights

• Over 14 million children attend summer day camps each year.
• Camps are well positioned to help children meet physical activity guidelines.
• This study evaluates a multi-summer physical activity intervention in 20 camps.
• Without intervention, children achieve high amounts of physical activity at camp.
• Efforts should focus on making summer day camps accessible to all children.

## 1 Introduction

National and international physical activity guidelines recommend all children (6–11 years old) accumulate 60 min per day (60 min/d) of moderate-to-vigorous physical activity (MVPA) (Health UDo et al., 2008, Tremblay et al., 2011, Health AGDo, 2014). However, the majority of children, both here in the U.S. and internationally, do not meet these recommendations (Troiano et al., 2008, Cooper et al., 2015). In an effort to improve the health and well-being of US children, some of the largest youth-based organizations – such as the YMCA of USA and the Boys and Girls Clubs of America – have adopted standards that align with the national physical activity guidelines calling for ‘full-day programs’, such as Summer Day Camps (SDCs), to provide children with opportunities to achieve 60 min/d of MVPA while in attendance.

SDCs present an ideal setting to increase children’s physical activity given their reach, with more than 5000 SDCs serving more than 14 million youth per year in the US (Association AC, 2014). Further, SDCs operate during the 3-months of the year where physical activity opportunities may be fewer as children are absent from the 9-month school-day environment with more regular physical activity opportunities (e.g., commute to school, recess, physical education, classroom breaks etc.) (Brazendale et al., 2017, Jago and Baranowski, 2004). This is particularly important as research has shown that during the 3-months of summer children exhibit accelerated weight gain (Moreno et al., 2013, von Hippel and Workman, 2016) and losses in fitness (Carrel et al., 2007, Gutin et al., 2008) compared to the 9-month school year. For these reasons, interventions during periods of time when children are at an increased risk of adverse health outcomes, such as summer, are of paramount importance (Weaver et al., 2018:).

Few interventions have been conducted in SDCs. Of those that have, they tend to focus or tailor toward a specific population (e.g., overweight/obese children (George et al., 2016), adolescent girls (Baranowski et al., 2003), children with autism spectrum disorder (Schenkelberg et al., 2015), have been limited by sample size (Huelsing et al., 2010), or have not used an objective measure of physical activity (Weaver et al., 2014). Given the importance of youth achieving 60 min/d of MVPA and SDCs recent adoption of guidelines for MVPA, there is a need to evaluate interventions designed to assist SDC providers in achieving this goal.

The objective of this study was to evaluate the effectiveness of a physical activity intervention at increasing the percentage of children meeting the 60 min/d MVPA guideline while in attendance. This study is reported in accordance with the Transparent Reporting of Evaluations with Nonrandomized Designs (TREND) statement (Des Jarlais et al., 2004).

## 2 Methods

### 2.1 Setting and participants

**Fig. 1:**
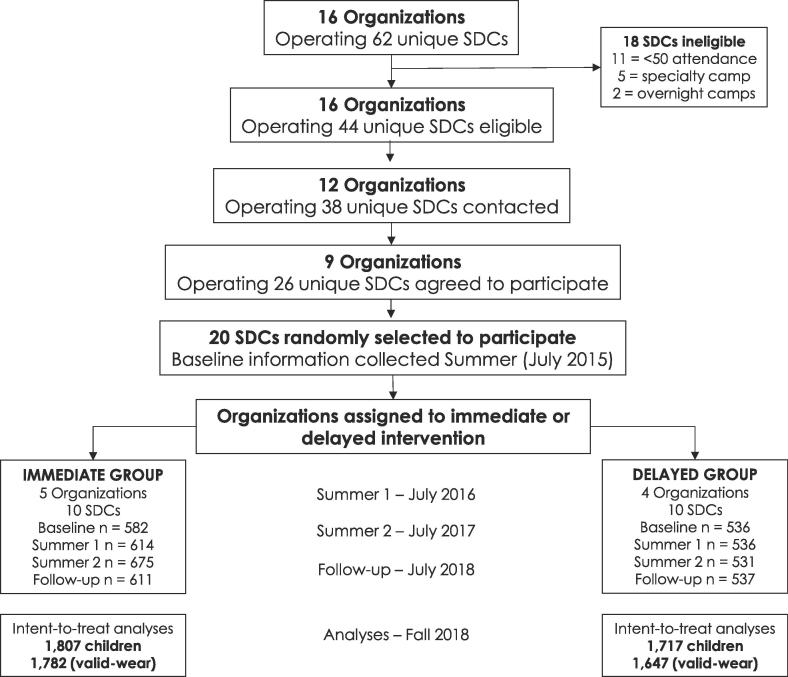
Flow chart of participants for recruitment, data collection, and analyses.

This study presents the physical activity outcomes from a 4-summer two-group, non-randomized trial in SDCs; Turn up the Healthy Eating and Activity Time (HEAT). Baseline (Brazendale et al., 2017) and first-year (Weaver et al., 2017) physical activity outcomes have been presented elsewhere. A total of 20 SDCs from nine different associations (e.g., YMCA’s, Boys & Girls Club of America, local parks and recreation commissions) in the Southeastern United States participated in this multi-summer intervention (Fig. 1). Details of SDC recruitment are described elsewhere (Brazendale et al., 2017, Weaver et al., 2017). In short, 62 SDCs within a 2-hour drive from the university were invited, through telephone and e-mail communication, to be part of the intervention. Eligibility criteria for SDC participating in the intervention consisted of a reported child enrollment of ≥50 children (based on previous summers enrollment) and classification as a non-specialized SDC (i.e., sport-specific camps, residential camps, academic camps). SDCs were defined as operating Monday-Friday, for at least 8 h per day during the hours of 8:00_AM_ to 6:00_PM_ and providing a mixture of activities each day from organized PA games and water-based activities, to arts and crafts and reading time. All attending children (≤12-years old), staff and SDC site leaders were eligible to participate in this study, however, children that were unable to engage in physical activity without an assistive device (e.g., wheelchair, supportive walking device) were excluded from measurement. Table 1 details the characteristics of the SDCs. The majority of the 20 SDCs were situated in urban- and suburban-based communities. Table 2 reports the demographic breakdown and accelerometer wear information for children included in this study. All study procedures were approved by the University of South Carolina’s institutional review board.

**Table 1:** Characteristics of participating summer day camps (SDCs) by intervention group.

SDC Characteristics	All Summers (2015–2018)			
	Immediate SDCs		Delayed SDCs	
Mean Daily SDC Attendance (±SD)	46.2	(18.4)	51.1	(24.9)
Mean Physical Activity Space in thousand’s ft^2^ (±SD)				
Indoor	10.4	(5.1)	13.1	(4.9)
Outdoor	172.2	(180.4)	259.4	(170.1)
Location (n)				
School	2		3	
Faith/church	3		0	
Community	2		7	
Other^a^	3		0	
Mean Temperature in degrees Fahrenheit (±SD)				
High	93.5	(4.6)	92.2	(4.6)
Low	73.8	(4.1)	73.4	(4.3)

^a^Strip mall, military base.

**Table 2:** Child-level characteristics by immediate and delayed intervention groups of children who attended SDC.

**Child Characteristics**	Summer 2015				Summer 2016				Summer 2017				Follow-up Summer 2018				All Summers (2015–2018)			
	IMM.		DEL.		IMM. *		DEL.		IMM. *		DEL.*		IMM.		DEL.		IMM.		DEL.	
Race/Ethnicity																				
% non-Hispanic White	37.3		15.2		37.5		17.7		37.2		16.5		37.9		17.1		37.5		16.6	
% African American	55.6		79.2		56.5		78.9		56.7		79.6		55.1		79.0		56.0		79.2	
% Other	7.1		5.6		6.0		3.5		6.1		3.9		7.0		3.9		6.5		4.2	
% Girls	49.6		43.9		51.9		36.5		49.4		41.5		51.9		42.1		50.7		41	
Age in Years (Mean, ±SD)	7.9	±1.8	8.0	±1.8	8.0	±1.7	7.8	±1.9	7.7	±1.7	7.7	±1.8	7.8	±1.8	7.7	±1.8	7.9	±1.7	7.8	±1.8
Children wearing accelerometer (n)	582		536		614		536		675		531		611		537		1,807		1,717	
Children meeting accelerometer wear inclusion criteria (n)^a^	576		512		597		517		664		510		597		509		1,782		1,647	
Number of valid wear days per child (Mean, ±SD)	2.7	±1.0	2.8	±1.0	2.9	±1.0	2.8	±1.0	2.6	±1.1	2.6	±1.0	2.4	±1.0	2.2	±1.0	2.7	±1.1	2.6	±1.0
Hours of wear time (Mean, ±SD)	8.3	±1.6	8.4	±1.8	8.3	±1.6	8.0	±1.9	7.9	±1.7	7.9	±1.8	8.3	±1.7	7.9	±1.9	8.2	±1.7	8.1	±1.9
Minutes of MVPA at SDC (Mean, ±SD)^a^	83.5	±13.2	95.6	±18.2	96.7	±7.5	94.9	±21.4	86.8	±12.7	85.7	±12.3	83.0	±13.6	87.5	±12.1	87.5	±13.2	90.9	±17.1

IMM. = Immediate Intervention Group; DEL. = Delayed Intervention Group, SDC = Summer Day Camps.

^a^Accelerometer wear inclusion criteria ≥ 240 min per camp day. Number represents unique children who may or may not have attended multiple summers.

*Intervention occurred during summer.

### 2.2 Study design and intervention group assignment

This study was designed as a multi-summer healthy eating and physical activity two-group delayed intervention with a follow-up phase. Evaluation of maintenance of the intervention after the removal of research personnel/support/training was an important feature of the study. In short, the intended study design was a matched-pair group randomized controlled trial based on initial baseline data (e.g., physical activity levels, foods provided, child demographics) (Beets et al., 2014). Unfortunately, due to changes in third party food providers (a key intervention component for the healthy eating aspect of the trial) contracted by SDC associations it was not possible to randomly assign SDCs that would result in comparable groups at initial baseline (summer 2015). Therefore, the authors decided to equally divide the 20 SDCs into two groups of 10 SDCs based upon an obvious separation of participating food providers for the SDCs. This split formed the basis allocating 10 SDCs to receive two summers of the physical activity intervention (immediate; 2016 and 2017) and 10 SDCs to receive a single summer (delayed; 2017) of the physical activity intervention. Summer of 2018 served as the follow-up no intervention summer.

### 2.3 Intervention

The intervention was a multi-component, capacity-building physical activity (and healthy eating) intervention aligning with previous large scale interventions targeting changes in children’s physical activity and diet in outside-of-school time settings at the program level (Weaver et al., 2017, Beets et al., 2016). The aim of this intervention was to increase physical activity levels of children in attendance was guided by two interrelated frameworks: Strategies To Enhance Practices (STEPs) (Beets et al., 2014) and the Theory of Expanded, Extended, and Enhanced Opportunities (TEO) (Beets et al., 2016).

#### 2.3.1 Strategies to enhance practices (STEPs)

The STEPs framework incorporates principles of adaptive intervention, community-based participatory research, and systems framework. Conceptually, the STEPs framework was designed as a multi-step, adaptive process where foundational programmatic components essential to increasing children’s physical activity are addressed in outside-of-school-time programs (e.g., afterschool program, summer day camp). Modifiable components of a program are identified and adapted incorporating aspects of individual-tailoring at the program-level to address the resources and constraints of each SDC. Finally, STEPs is an adaptive and flexible framework that allows SDCs to enter, and benefit from, a continuum of support regardless of the current status of a SDCs physical activity promoting environment (Beets et al., 2014). For the intervention, STEPs was the overarching guiding framework through which each element of TEO was implemented. Further details on the STEPs framework, and the design and overview of the trial can be found elsewhere (Beets et al., 2014).

#### 2.3.2 Theory of expanded, extended, and enhanced opportunities (TEO)

The foundational components and core physical activity-promoting strategies incorporated by the SDCs were guided by the TEO. The theory postulates that the primary mechanism for increasing children's accumulation of physical activity is through the provision of opportunities to be physically active through three mechanisms: 1) *extending* physical activity opportunities (i.e., allocating extra time for existing physical activity opportunities), 2) *expanding* physical activity opportunities (i.e., adding a new physical activity opportunity), and 3) *enhancing* physical activity opportunities (i.e., modifying existing physical activity opportunities to maximize the amount of physical activity youth accumulate) (Beets et al., 2016).

In the spring (April-May) of each intervention summer researchers responsible for delivering the intervention met with SDC staff and the SDC site leader to deliver a 2-hour physical activity promotion training guided by the three mechanisms of TEO (expanding, extending, enhancing). Specific strategies included working with the SDCs to 1) expand PA opportunities by integrating PA into enrichment time through short activity breaks (e.g., energizers, brain breaks, etc.) or by exchanging inactive field trips (e.g., movie theatre) for more active field trips (e.g., pool, park), 2) schedule a minimum of three hours of physical activity time each day (extend), and 3) enhance the amount of activity children accumulated during existing physical activity opportunities using the evidence-based LET US Play principles (Weaver et al., 2013, Brazendale et al., 2015). During each training, research staff worked directly with SDC staff and site leaders to help identify, modify and generate new ideas tailored towards the individual programmatic elements of the SDC (e.g., daily schedule, staff, resources, space etc.). In addition to the 2-hour training, and prior to primary outcome data collection, trained research staff visited intervention SDCs on two non-consecutive unannounced days for booster sessions in the month of June (summer 2016, 2017). The purpose of the booster sessions was to do a 60-minute walkthrough of the SDC with the SDC site leader to observe the implementation of the various strategies related to TEO (extend, expand enhance) that was covered in the 2-hour training in the spring. At the end of the walkthrough, 30 min was allocated for research staff to meet with SDC staff and the site leader to discuss and problem solve any challenges they had faced when trying to implement some of the strategies targeted in their initial 2-hour training. SDC trainings and booster sessions were not implemented in spring 2018 prior to the follow-up summer.

### 2.4 Measures

#### 2.4.1 Main outcome evaluation – Physical activity assessment

Consistent with prior protocols (Brazendale et al., 2017, Weaver et al., 2017), and in line with previous studies capturing children’s free-living physical activity in outside-of-school time settings (Weaver et al., 2017, Beets et al., 2009, Gortmaker et al., 2012), children’s physical activity was assessed using ActiGraph accelerometers (version GT3X-BT and Link – Shalimar, FL). Each summer, data collection took place during the months of July and August with each individual SDC visited on four non-consecutive unannounced days (Monday-Thursday). On data collection days, trained research staff placed an accelerometer on the child’s non-dominant wrist upon arrival at the SDC (Time ON). Before leaving the SDC, research staff removed the accelerometer from the child (Time OFF). Time ON and Time OFF was recorded to the nearest minute. Children were allowed to participate in all regularly scheduled activities for the day including wearing the accelerometer during off-site field trips and water-based activities. Non-dominant wrist-based cutpoints associated with children's MVPA (≥530 counts/5 s epoch) (Chandler et al., 2015) were used to distill the data. A valid day of wear was considered greater than 240 min (Time ON minus Time OFF) (Brazendale et al., 2017) with removal of non-wear time identified as consecutive zeros for 30 min or more (Cain et al., 2013). Prior to accelerometer placement, assent was obtained from the child on every day of data collection. An opt-out protocol was used to obtain passive consent from participating children's parents, however, prior to data collection SDCs provided parents with informational fliers about data collection procedures and instructions on how to opt out of the study. In conjunction with accelerometer placement, participating child-level demographics (i.e., age, race, sex) were collected by pencil and paper by research staff. Indoor and Outdoor space was measured using a Lufkin Pro-Series 9999-ft Measuring Wheel. Temperature was recorded every data collection day from Weather Underground (www.wunderground.com/) and logged in an Excel file.

#### 2.4.2 Process evaluation – Implementation of TEO

On the same four unannounced visits to the SDC for physical activity assessment, trained research staff collected process evaluation data. The implementation of TEO components was measured using the System for Observing Staff Promotion of Activity and Nutrition (SOSPAN) (Weaver et al., 2014) – a direct observation tool designed and validated for use in outside-of-school time settings such as SDCs. Details of SOSPAN procedures are described in more detail in the published first-year outcomes (Weaver et al., 2017). In short, SOSPAN protocol requires a person to rotate through pre-defined ‘target areas’ (i.e., pre-identified areas of the SDC) while completing continuous scans of the environment from the beginning (morning arrival; ~7:30_AM_) to the end (afternoon departure; ~6:00_PM_ of SDC day. Prior to every summer research staff were trained in SOSPAN procedures taking part in classroom sessions, including video analysis and a thorough review of protocols. Following this, staff spent approximately 6 days (3 h/day) over a 3-week period conducting field-based trainings at local SDCs not participating in the study. In accordance with previously established direct observation protocols, inter-rater agreement and reliability was assessed (McKenzie et al., 1992). Inter-rater agreement criteria were set at *>* 80% using interval-by-interval agreement, and inter-observer reliability was estimated via the percent agreement and weighted kappa (κw). Reliability was checked daily to identify any disagreements. To ensure acceptable reliability and prevent ‘observer drift’, operational definitions of variables with low reliability (<90% agreement) were discussed. The percent agreement for all summers ranged from 92.8 to 99.8% and κw ranged from 0.50 to 0.94.

#### 2.4.3 TEO – Expand

The SOSPAN instrument was used to capture any instances where physical activity opportunities were expanded (i.e., a new physical activity was introduced). As part of the intervention, SDCs were encouraged to 1) introduce activity breaks – defined as brief activities (3 to 5 min), led by a staff member that occur during an otherwise sedentary time in a non-active setting (e.g., during enrichment taking place in a classroom) and/or 2) replace non-active field trips (e.g., movies) with active field trips (e.g., parks/playground, pools). Field trips were defined as any instance when children and counselors left the normal SDC location. In this instance, SOSPAN did not take place during the field trip, rather research staff accompanied SDCs on field trips and noted the location, duration, and specific details of the activities on the field trip.

#### 2.4.4 TEO – Extend

In order to capture and quantify the amount of time SDCs allotted for physical activity and other activities (e.g., enrichment, meals/snacks) two methods were used. First, on each observation day a schedule was collected from the site leader of the SDC. Second, as part of the SOSPAN process (Weaver et al., 2014), data collectors indicated the context they were observing at the beginning of each scan. The SDC contexts included: meal or snack, enrichment, academics, physical activity, or other (transition, bathroom/water break, camp assembly, etc.). Consistent with previous protocols (Weaver et al., 2017), the percent of scans completed within a given context was used to identify the percentage of a SDCs' schedule allocated for each context. The percentage of scans in each context was cross-referenced with the hard-copies of the SDC schedules obtained from the site leaders to identify discrepancies. If discrepancies occurred the observed SOSPAN time was used as the indicator of the amount of time spent in particular context (Weaver et al., 2017).

#### 2.4.5 TEO – Enhance

SOSPAN was used to capture implementation (percent of scans) of the following 12 LET US Play principles (Weaver et al., 2013) during observed physical activity opportunities: children waiting in line for a turn, children eliminated from activity, children waiting for activity to start (i.e., idle time), staff withholding physical activity as punishment, staff disciplining children with physical activity, small sided-games being played, staff actively engaged in activity, staff verbally encouraging activity, staff leading an activity, choice of two or more activities offered, girl's provided with their own physical activity opportunities, and staff giving instructions on how to play games. In accordance with prior analyses (Weaver et al., 2017) the distribution of each LET US Play principle was tertiled based on the 33rd and 66th percentile at baseline (summer 2015). SDCs were assigned a one (*<*33rd centile), two (33rd to 66th centile), or three (*>*66th centile) for each principle. Where necessary, LET US Play principles were reverse coded and scores were summed to represent an overall LET US Play implementation score for each summer (possible range of scores = 12–36 points).

### 2.5 Statistical analysis

In accordance with previously established protocols, children with at least one valid day of accelerometer wear during any measurement summer (baseline to follow-up) were included in the analyses (Weaver et al., 2017), and a-prior power calculations were performed using Optimal Design HLM Software (v.2.0) and an anticipated average of 50 children per SDC, and a total of 20 ASPs (estimated total sample size for children = 1000), the study had a power of 0.80 to detect a 16% increase in the outcome (Beets et al., 2014). Descriptive statistics (means, standard deviations, percentages) and the percent of boys and girls meeting the 60 min/d guidelines and a group-by-time interaction was computed prior to the main analysis. For the main analysis, separate (boys and girls) multi-level mixed effects logistic regression models were used to estimate the impact of the intervention on the percentage children meeting the 60 min/d MVPA guideline. All models accounted for the nested nature of the data with days nested within children nested within SDC and models employed full-information maximum likelihood estimators to account for missing activity data and included age, race and SDC size. Using the multi-level mixed effects models presented above, the following analyses were conducted for both intervention groups combined using the ‘*contrast’* command in STATA which allows for comparisons of effects in multi-level models to be examined (Keppel and Wickens, 2004). 1) Intervention versus Baseline summers, 2) Intervention vs. Follow-up summers, and 3) Follow-up versus Baseline summers.

### 2.6 Process evaluation

At the SDC level, correlations assessed the extent to which the change in a TEO component impacted the change in the percent of children the 60 min/d MVPA guideline. This was based on change from the previous summer. All analyses were intent-to-treat and conducted using STATA (v.14.2, College Station TX).

## 3 Results

### 3.1 Outcome Evaluation

Across the 20 SDCs 9792 child observation days were collected from 3524 unique children of which, 9350 (95.5%) were valid accelerometer wear days with ≥ 240 min in attendance (n = 3429 unique children). Across the 20 SDCs, the percent of children returning to SDC for a 2nd, 3rd, or 4th summer was 24%, 4%, and 1%, respectively. The mean MVPA estimate across all summers and SDCs was 89.2 min/day (±22.5) (Table 2). Table 3 presents data by SDC, and SDC association on the percent of boys and girls attaining 60 min/d of MVPA each summer and the change from baseline to follow-up. There was no significant group-by-time effect of receiving the intervention for 2 years (immediate group) versus 1 year (delayed group). Table 4 displays boys’ and girls’ model-derived odds of meeting the 60 min/d MVPA guideline. The likelihood of meeting the 60 min/d MVPA guideline was not different during intervention versus baseline summers for boys or girls. Girls and boys were 3.5 (95CI = 1.5, 8.1) and 3.7 (95CI = 1.6, 8.4) times more likely to meet the 60 min/d guideline during intervention summers versus follow-up, respectively. Boys were 2.8 (95CI = 1.2, 6.4) times more likely to meet the guideline at baseline versus follow-up.

**Table 3:** Percent of boys and girls meeting national daily physical activity recommendations (60 min/d) by Summer Day Camp (SDC).

Intervention Group	SDC	SDC Association	Percent of Attending Children Meeting 60 min/d of MVPA*							
			Boys				Girls			
			Baseline^a^	Intervention Summers	Follow-up	Δ	Baseline^a^	Intervention Summers	Follow-up	Δ
**Immediate** (2 summers of intervention)	1	A	88.9	85.1	81.3	−7.6	77.2	78.4	64.5	−12.7
	2		79.4	83.8	71.8	−7.6	70.3	79.9	74.3	4
	3		92.1	86.3	76.9	−15.2	77.1	74.3	67.4	−9.7
	4		64.7	93.4	80.6	15.9	51.9	80.8	16.7	−35.2
	5	B	41.1	64.3	54.1	13.0	29.9	60.0	44.2	14.3
	6		75.6	81.3	78.4	2.8	61.6	85.4	85.3	23.7
	7	C	84.3	94.5	96.0	11.7	77.6	86.9	87.7	10.1
	8	D	88.9	82.3	82.1	−6.8	79.7	78.5	79	−0.7
	9	E	86.9	90.4	72.5	−14.4	83.1	85.9	68.6	−14.5
	10		84.3	88.7	93.2	8.9	85.4	75.4	72.2	−13.2
		**Average**	78.6	85.0	78.7	0.1	69.4	78.5	66.0	−3.4

**Delayed** (1 summer of intervention)	11	F	73.3	100.0	73.9	0.7	78.5	84.6	69.2	−9.3
	12		77.0	73.2	70.4	−6.6	59.2	68.2	65.7	6.6
	13		67.9	61.7	63.0	−4.8	64.0	61.0	36.8	−27.2
	14	G	95.2	80.4	95.0	−0.2	93.7	83.3	92.9	−0.8
	15		79.9	77.0	89.7	9.8	76.5	84.2	83.8	7.3
	16		90.9	68.2	70.7	−20.2	87.1	63.6	71.7	−15.4
	17		94.5	91.3	68.8	−25.7	76.7	67.1	79.3	2.6
	18		90.0	93.9	94.9	4.9	69.5	75.8	91.1	21.7
	19	H	78.4	92.0	81.8	3.5	61.0	73.1	53.8	−7.2
	20	I	79.0	68.4	70.3	−8.7	79.4	68.8	91.9	12.5
		**Average**	82.6	80.6	76.6	−4.7	74.5	73.0	73.6	−0.9
**All**		**Average**	80.6	82.8	78.3	−2.3	72.0	75.8	69.8	−2.2

*All estimates are means from raw data and exclude children who < 240 min/day of accelerometer wear time.

MVPA = Moderate-to-vigorous physical activity.

Δ Represents change from Baseline to Follow-up.

^a^Baseline represent all summers prior to intervention summer.

**Table 4:** Boys' and girls' model-derived odds of meeting 60 min/d of moderate-to-vigorous physical activity (MVPA).

Group	Odds of Meeting 60 min/d MVPA Guideline^a^											
	Analysis 1				Analysis 2				Analysis 3			
	Intervention vs. Baseline*				Intervention vs. Follow-up				Baseline* vs. Follow-up			
	OR	SE	95%CI		OR	SE	95%CI		OR	SE	95%CI	
Girls	1.80	0.81	0.74,	4.35	**3.48**	1.50	**1.49,**	**8.11**	1.93	0.86	0.81,	4.60
Boys	1.33	0.57	0.58,	3.07	**3.69**	1.55	**1.62,**	**8.40**	**2.77**	1.17	**1.21,**	**6.36**
All^b^	1.41	0.48	0.73,	2.75	**3.62**	1.11	**1.98,**	**6.61**	**2.56**	0.80	**1.39,**	**4.73**

Bolded values indicate statistical significance (p < 0.05). OR = Odds Ratio, SE = Standard Error.

^a^Model derived estimates control for age, camp attendance size, and race.

*Baseline represent all summers prior to intervention summer.

^b^Sex controlled for overall analysis.

### 3.2 Process evaluation – TEO

Table 5 represents the implementation of extended, expanded and enhanced physical activity opportunities by SDCs groups. The majority of the extension in physical activity time was apportioned to free-play (unstructured) physical activity. During intervention summers, SDCs varied in their implementation of additional active field trips (expanded) but increased the number of physical activity breaks (expanded) observed during camp days (i.e., increase in SOSPAN Scans on intervention versus baseline summers). An average of 5 SDCs enhanced physical activity opportunities during intervention summers versus baseline summers by increasing their LET US Play Index score. Comparing follow-up to baseline, 8 SDCs (4 immediate intervention, 4 delayed intervention) increased their LET US Play Index score (Table 5). At the SDC level, the TEO component of extension had the largest correlation (r = 0.40) with change in the percent of children meeting the 60 min/d guideline compared to expansion (r = 0.12) and enhancement (r = 0.07).

**Table 5:** Implementation of Expanded, Extended, and Enhanced Physical Activity Opportunities at Summer Day Camps (SDCs).

TEO Component	Immediate					Delayed				
	Summer 2015	Summer 2016 *	Summer 2017 *	Summer 2018 (Follow-up)	Δ	Summer 2015	Summer 2016	Summer 2017 *	Summer 2018 (Follow-up)	Δ
**Expand**										
Total field trips (n)	13	12	12	12	−1	9	5	3	8	−1
Active field trips (n)	8	8	10	9	1	3	2	2	5	2
Number of SOSPAN scans of Activity Breaks ǂ	9	113	114	75	66	17	77	148	25	8

**Extend**										
Average daily SDC operating time (min)	639	675	636	647	8	617	654	651	635	18
% of Daily Schedule allocated for:										
Meal or Snack	16.5	14.4	17.0	15.3	−1.2	16.6	15.2	14.9	14.3	−2.3
Enrichment	46.9	42.1	42.8	43.9	−3.0	36.3	42.2	37.0	34.7	−1.6
Academics	2.3	1.7	5.0	2.7	0.4	3.5	1.4	3.8	2.3	−1.2
Other^a^	9.2	6.7	6.3	8.0	−1.2	9.5	6.3	11.5	12.9	3.4
Physical Activity	25.2	35.1	30.1	30.1	4.9	34.1	34.9	35.0	35.8	1.7
- Free Play	12.0	15.3	15.1	15.8	3.8	19.3	14.3	15.9	21.2	1.9
- Organized	13.2	19.8	15.0	14.3	1.0	14.8	20.6	19.1	14.6	−0.2

**Enhance**										
LET US Play Index Score	24.3	24.9	25.0	25.9	1.6	24.2	23.2	25.4	25.1	0.9
Number of SDCs increasing LET US Play index score^b^		4	5	6	4		3	7	4	4

TEO = Theory of Expanded, Extended, and Enhanced Opportunities.

*Intervention occurred during summer.

Δ Represents change from Baseline.

^a^Bathroom, water breaks, idle time etc.

^b^Compared to previous summer LET US Play Index score of SDC increased by ≥1.0.

^ǂ^Note: Multiple SOSPAN scans can capture 1 Activity Break.

## 4 Discussion

This study is one of the first to investigate the effectiveness of a multi-summer SDC intervention at increasing the percentage of children meeting the 60 min/d of MVPA guideline while in attendance. The results of this physical activity intervention demonstrate it was not successful at increasing the percent of children meeting this guideline. Specifically, the likelihood of boys and girls meeting the guideline did not increase during intervention summers compared to baseline summers. Although the outcomes of this intervention are disappointing, there are still several key lessons learned as a result of conducting this study.

The SDC MVPA estimates reported in this study (~89 min/d MVPA) are notably higher than other studies reporting MVPA estimates of children attending SDCs (range 37–58 min/SDC/day) (Baker et al., 2017, Beets et al., 2011). This could be due to a higher mean daily wear time in this study (504 vs. 340 min/d) (Beets et al., 2011) or the different accelerometer placement site (i.e., non-dominant wrist versus hip) (Baker et al., 2017). Nonetheless, it is important to recognize that all SDCs in this study provided boys and girls with a substantial amount of MVPA across all measurement summers. This is reflected in the fact that for any given summer, approximately >70% of boys or girls were accumulating 60 min of MVPA for the hours that they were in attendance. Perhaps this is not surprising as SDCs allocated large proportions of their daily schedule (25%–35% of daily SDC time) for physical activity opportunities on both intervention and baseline summers. This notion is supported by the moderate positive correlation between the TEO component of extension (i.e., increase time for physical activity opportunities) and the change in the percent of children meeting the guideline (r = 0.40). Providing opportunities for children to be active (versus no opportunities), therefore, is an effective strategy for children to accumulate MVPA. This finding aligns with several school-based studies that show children are more active on days when provided one or several activity opportunities, versus fewer or none at all (Cardon et al., 2008, Carlson et al., 2015, Tudor-Locke et al., 2006, Brusseau et al., 2011). Thus, incorporating TEO as a framework to guide future summer staff trainings to enhance physical activity is recommended. Nonetheless, it appears that a SDC, in and of itself, is an intervention, which is consistent with recent behavioral frameworks (Brazendale et al., 2017;14(1):) that suggest that children’s obesogenic behaviors – such as physical activity – are beneficially regulated (e.g., increase in physical activity) when the child is exposed to a ‘structured’ day, such as the presence of a SDC.

SDCs possess great potential as a setting where children can attain health-enhancing levels of physical activity, with minimal support, during a time of year (summer) identified as detrimental to children’s health (Weaver et al., 2018:). Although SDCs serve over 14 million children per year, there is still a large proportion of children who do not have access to this setting due to barriers such as cost. In their recent Camper Enrollment Report, the largest provider of SDCs in the U.S., the American Camp Association (ACA), revealed that 70% of attendees were non-Hispanic white, from middle-income households, and the number of requests from families for scholarships and reduced fees had increased from the previous year (Camper and Enrollment, 2017). In light of this, public health practitioners should focus efforts on making summer day camps accessible for all children and families in the U.S.

Exploring the summer-to-summer variability in MVPA estimates of children attending SDCs, and the fluctuations of estimates within- and between-SDCs from the same SDC association highlights the complex and ever-changing nature of the SDC setting. A clear example of this is presented in Table 3 where SDCs continued to improve from baseline to follow-up summers (SDC 7, Association C), some improved from receiving the intervention for one summer (SDC 11, Association F), some remained relatively unchanged (SDC 8, Association D), some decreased MVPA despite receiving the intervention (SDC 16, Association G), and some increased MVPA estimates during the follow-up in comparison to baseline summers despite receiving no intervention for the last summer (SDC 10, Association E; SDC 18, Association G). The variability in MVPA and responsiveness to the intervention within an SDC association suggests that children's MVPA and programmatic structure changes are largely driven at the SDC-level, rather than at the association level, and could be severely impacted by other factors not captured in this study (e.g., staff turnover). Nonetheless, in light of this, future SDC intervention efforts should primarily be directed to the individual SDC and the persons responsible for the day-to-day operations of the SDC and their staff. Additionally, future work could examine any lagged or wash-out effects of intervention trainings with staff, or explore the role continued (but reduced intensity) booster sessions can play in maintaining intervention implementation.

Despite a lack of statistically significant outcomes, there are numerous strengths of this study. It is one of the first to evaluate the effectiveness of a multi-year physical activity intervention in a large sample of traditional SDCs serving thousands of children. This study incorporated a theory-based framework designed to meet the individual demands and resources of SDCs, included a follow-up phase to assess maintenance of the intervention, and used an objective measurement of children’s MVPA. There are limitations to this study that must be noted. First, this study was not a randomized trial as planned due to circumstances outside the control of the investigative team. Second, the SDCs evaluated in this intervention are only representative of SDCs in the southeastern U.S., largely serving urban- and suburban-dwelling children, and thus, generalizability of these findings may be limited to other settings (e.g., rural SDCs). Third, MVPA estimates presented in this study are for time-in-attendance only, limiting the ability to distinguish the contribution of SDCs to total daily MVPA.

In conclusion, SDCs are a promising setting where children can achieve health-enhancing levels of daily physical activity during a time of year – summer – identified as a ‘critical window’ for children’s health (Weaver et al., 2018:). Although the results of this intervention demonstrate that the current approach was not successful at increasing children’s MVPA, a large proportion of the boy and girls were already meeting national daily physical activity recommendations at baseline (Brazendale et al., 2017) and successive summers. The implications of this are far-reaching. Given their widespread presence across the U.S. and their typical hours (7:00 am to 6:00 pm) and length (8–10 weeks) of operation over summer, SDCs offer an ideal setting to engage children with a health-enhancing environment during months of the year when children are at a greater risk of negative health outcomes such as accelerated weight-gain (Moreno et al., 2013) and losses in cardiorespiratory fitness (Carrel et al., 2007). Thus, public health practitioners and policymakers should focus efforts on making SDCs an accessible and prevalent setting for all children in the U.S.

## CRediT authorship contribution statement

**Keith Brazendale:** Formal analysis, Writing - original draft. **Michael W. Beets:** Conceptualization, Methodology, Funding acquisition. **R. Glenn Weaver:** Supervision, Writing - review & editing. **Gabrielle M. Turner-McGrievy:** Writing - review & editing. **Justin B. Moore:** Writing - review & editing. **Jennifer L. Huberty:** Writing - review & editing. **Dianne S. Ward:** Writing - review & editing.
